# Enhanced prevention on postoperative atrial fibrillation by using anti-inflammatory biodegradable drug patch

**DOI:** 10.1093/rb/rbaf040

**Published:** 2025-05-14

**Authors:** Pengcheng Yu, Weiqi Lu, Huaxin Sun, Chengchen Huang, Xiaolin Zhou, Yuxing Wang, Zhen Zhang, Guosheng Fu, Hanxiong Liu, Kefeng Ren, Xia Sheng

**Affiliations:** Key Laboratory of Cardiovascular Intervention and Regenerative Medicine of Zhejiang Province, Department of Cardiology, Sir Run Run Shaw Hospital, Zhejiang University School of Medicine, Hangzhou 310016, China; Engineering Research Center for Cardiovascular Innovative Devices of Zhejiang Province, Hangzhou 310016, China; MOE Key Laboratory of Macromolecular Synthesis and Functionalization, International Research Center for X Polymers, Department of Polymer Science and Engineering, Zhejiang University, Hangzhou 310058, China; Department of Cardiology, The Third People’s Hospital of Chengdu, Affiliated Hospital of Southwest Jiaotong University, Chengdu 610014, China; Key Laboratory of Cardiovascular Intervention and Regenerative Medicine of Zhejiang Province, Department of Cardiology, Sir Run Run Shaw Hospital, Zhejiang University School of Medicine, Hangzhou 310016, China; Engineering Research Center for Cardiovascular Innovative Devices of Zhejiang Province, Hangzhou 310016, China; Department of Cardiology, The Third People’s Hospital of Chengdu, Affiliated Hospital of Southwest Jiaotong University, Chengdu 610014, China; Key Laboratory of Cardiovascular Intervention and Regenerative Medicine of Zhejiang Province, Department of Cardiology, Sir Run Run Shaw Hospital, Zhejiang University School of Medicine, Hangzhou 310016, China; Engineering Research Center for Cardiovascular Innovative Devices of Zhejiang Province, Hangzhou 310016, China; Department of Cardiology, The Third People’s Hospital of Chengdu, Affiliated Hospital of Southwest Jiaotong University, Chengdu 610014, China; Key Laboratory of Cardiovascular Intervention and Regenerative Medicine of Zhejiang Province, Department of Cardiology, Sir Run Run Shaw Hospital, Zhejiang University School of Medicine, Hangzhou 310016, China; Engineering Research Center for Cardiovascular Innovative Devices of Zhejiang Province, Hangzhou 310016, China; Department of Cardiology, The Third People’s Hospital of Chengdu, Affiliated Hospital of Southwest Jiaotong University, Chengdu 610014, China; Key Laboratory of Cardiovascular Intervention and Regenerative Medicine of Zhejiang Province, Department of Cardiology, Sir Run Run Shaw Hospital, Zhejiang University School of Medicine, Hangzhou 310016, China; Engineering Research Center for Cardiovascular Innovative Devices of Zhejiang Province, Hangzhou 310016, China; MOE Key Laboratory of Macromolecular Synthesis and Functionalization, International Research Center for X Polymers, Department of Polymer Science and Engineering, Zhejiang University, Hangzhou 310058, China; Key Laboratory of Cardiovascular Intervention and Regenerative Medicine of Zhejiang Province, Department of Cardiology, Sir Run Run Shaw Hospital, Zhejiang University School of Medicine, Hangzhou 310016, China; Engineering Research Center for Cardiovascular Innovative Devices of Zhejiang Province, Hangzhou 310016, China

**Keywords:** postoperative atrial fibrillation, inflammation, oxidative stress, drug-eluting patch, andrographolide

## Abstract

Postoperative atrial fibrillation (POAF) is the most prevalent form of secondary atrial fibrillation and increases the risk of adverse cardiovascular outcomes, such as stroke, heart failure and increased mortality. Herein, we designed an andrographolide (Andr)-loaded degradable polymer patch to deliver the drug directly to the atrial tissue for prevention of POAF. The sterile pericarditis (SP) rat model was adopted for highly relationship to clinical practice. The patch-released Andr effectively reduced the incidence of atrial fibrillation from 90 to 20%, and alleviated local atrial inflammation and oxidative stress *in vivo*, by using electrophysiological detection and histological analysis such as immunofluorescence, western blot and PCR. In HL-1 cells, we found the use of Andr-loaded patch could strongly inhibit the cell death, reactive oxygen species (ROS) generation and mitochondrial injury caused by LPS. Meanwhile, the use of Andr-loaded patch could effectively inhibited macrophages polarize towards M1. Mechanistically, we verified that the regulation of the cytoplasm and mitochondria Ca^2+^ and ROS dynamic balance was quite important both *in vivo* and *in vitro*. Our strategy proved by regulating the inflammatory microenvironment, ROS balance and Ca^2+^ homeostasis and the Andr-loaded atrial patch was effective for POAF in the SP rat model. The electrical signal of atrial stromal reentry in the case of this model was successfully mined, and the results of calcium channel were basically consistent with that of electrical signal channel. In addition, we have reported the infiltration and polarization of local inflammatory cells in the atrial of POAF at the tissue section level. Our study served as a new inspiration for the treatment of arrhythmic diseases and other ROS- and Ca^2+^- associated local illnesses.

## Graphical Abstract

**Figure:**
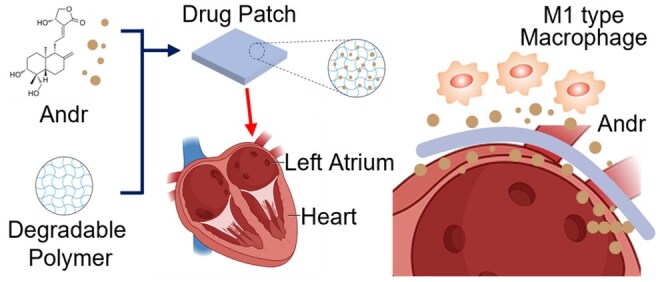

## Introduction

Postoperative atrial fibrillation (POAF), the most prevalent form of secondary atrial fibrillation (AF), often occurs within three days following surgery and increases the risk of adverse cardiovascular outcomes, such as stroke, heart failure and increased mortality [1, 2]. Research has extensively investigated POAF in both cardiac [3] and noncardiac [4] surgery contexts, with incidence rates of approximately 30% in the former and 0.4–15% in the latter. Emerging evidence suggests a significant role for inflammation and oxidative stress in the development of POAF [5].

Nanoparticles [6], hydrogels [7] and drug-loaded scaffolds [8] are effective methods for the sustained release of therapeutic agents to facilitate cardiac repair. However, given the dynamic nature of the heart, its position and shape can change with contraction and relaxation of the cardiac muscles, making accurate and localized injection of hydrogels into the atrial surface a significant challenge. Nanoparticles and scaffolds face similar problems. Correspondingly, patches are in the form of thin sheets, which are easy to fit to the surface of myocardial tissue, and are relatively stable and not easy to slide. Therefore, we fabricated a drug-loaded atrial patch that exhibited both degradability [9] and mechanical properties [10]. The patch was constructed using cross-linked poly(lactic acid)-poly(ethylene glycol)-poly(lactic acid) (PLA-PEG-PLA, PEL) triblock copolymer networks [11] and enabled direct *in situ* implantation following open-heart surgery. Both PEG- and PLA-based copolymers have been approved by the US Food and Drug Administration for biomedical applications owing to their excellent biocompatibility [12]. In this study, we employed andrographolide (Andr)-loaded patch approach to achieve precise drug delivery to the atrial region for POAF.

Andr, widely used in clinical treatments, is derived from the herb *Andrographis paniculata*, a staple in traditional Chinese medicine [13]. In cardiac research, Andr has shown efficacy in inhibiting the activation of cardiac fibroblasts and in protecting against aortic banding-induced cardiac hypertrophy by blocking the MAPK signaling pathway [14]. It has also been found to offer protective effects against MI induced by isoproterenol through the inhibition of L-type Ca^2+^ channel function and the enhancement of transient outward K^+^ currents, in addition to reducing adverse cardiac remodeling by activating Nrf2 signaling [15]. Previous studies have indicated Andr's role in preventing atrial fibrillation in a rapid atrial pacing rabbit model by regulating mitochondrial reactive oxygen species (ROS) production [16].

Rather than the other inflammatory model reported previously in a review [17], we selected the sterile pericarditis (SP) inflammation-related rat model, which exhibited significant Ca^2+^ mishandling and Ca^2+^ treatment heterogeneity, as well as increased susceptibility to Ca^2+^ transient (CaT) and action potential duration (APD) alternations [18]. The incidence of POAF is higher in cardiac surgery, including opening the pericardium, which may be due to direct mechanical injury. In addition, the characteristic of the SP model was local inflammation rather than the circulating inflammation, which was highly closed to clinical chest surgery and greatly consistent with our concept of local drug-loaded patch precision therapy. However, even in the absence of hemodynamic damage, a small amount of blood in the pericardium can induce POAF through inflammation and oxidative stress. When reactive oxygen species (usually H_2_O_2_ produced by white blood cells) cause lipid peroxidation of the atrial cell membrane and periatrial fat pad, oxidative stress occurs, leading to membrane rupture, mitochondrial dysfunction, intracellular Ca^2+^ overload and ultimately apoptosis and necrosis [19]. It is reported that in skeletal muscle atrophy, oxidative stress is closely related to mitochondrial dysfunction [20]. Thus, we speculated that Ca^2+^, inflammation and oxidative stress are the potential targets in POAF. Considering these, we chose the optical mapping in our research to detect the therapy effect of drug-loaded patch on the Ca^2+^-related phenotype and the change of APD. We established a connection between oxidative stress and electrophysiological- Ca^2+^ research through this detection method.

In this study, taking into consideration the critical role of inflammation and oxidative stress in the generation and development of POAF, we employed the drug-loaded patch approach to achieve precise drug delivery to the atrial region for POAF.

## Materials and methods

### Materials

Poly (ethylene glycol) (PEG, Mn = 2000), DL-lactide, dimethyldichlorosilane and 2-isocyanatoethyl methacrylate were purchased from Macklin (Shanghai, China). 2-Hydroxy-4-(2-hydroxyethoxy)-2-methylpropiophenone (I2959) and 4-methoxy phenol were obtained from Aladdin (Shanghai, China). Andr, stannous octoate (Sn(Oct)_2_) and rhodamine B isothiocyanate (RITC) were purchased from Sigma-Aldrich (USA). Phosphate buffered saline (PBS) was purchased from Sango Biotech (Shanghai, China). The deionized water (>18 MΩ·cm) used in all experiments was purified with a Millipore Milli-Q water purification system. All the materials were used as received without further purification.

### Synthesis and characterization of PEL triblock copolymers

For the synthesis of the PEL triblock copolymers, PEG was used as the initiator for the coordination-based ring-opening polymerization [21] of DL-lactide in the presence of Sn(Oct)_2_ as the catalyst. Initially, PEG was added to a 50 mL three-necked round-bottom flask and dried at 70°C for 1 h under vacuum. Then, 3.25 g of DL-lactide and 5 mg of Sn(Oct)_2_ (1 wt%) were added under a dry argon atmosphere and heated to 140°C for 4 h. To ensure complete melting of DL-lactide from the inner wall of the flask, three-necked bottles were heated every 30 min during the reaction. After DL-lactide condensation on the inner wall of the flask was nearly absent, 5 mg of 4-methoxyphenol (1 wt%) was added to terminate the reaction. Subsequently, 2-isocyanatoethyl methacrylate was dissolved in toluene and added in multiple portions under an argon atmosphere. The reaction was allowed to proceed for 20 min. After the completion of the reaction, the mixture was cooled to room temperature, dissolved in dichloromethane and precipitated in excess ethyl alcohol. The resulting product was then dried under vacuum at room temperature for 24 h.

The molecular structure, composition and molecular weight of the PEL triblock copolymer were determined using ^1^H NMR (AVANCE NEO 400, Bruker, Switzerland) and gel permeation chromatography (GPC, Waters 1515, Waters, USA).

### Fabrication of drug-loaded atrial patch

To prepare PEL atrial patches, we dissolved PEL, I2959 and Andr in acetone using a mass ratio of 1000:1:0.1. The PEL atrial patches were dried in 10 wt% acetone solutions in Teflon molds with dimensions of 3 × 3 cm^2^. Acetone was evaporated slowly at 25°C for 24 h, followed by a UV photocrosslinking process using an Intelli-Ray 400 UV oven for 150 s. After the film was removed, a relatively flat area of the patch was cut into patches measuring 2 × 2 cm^2^.

### Differential scanning calorimetry

Differential scanning calorimetry (DSC) (Discovery DSC25/TA, USA) was performed to determine the thermal properties of the patches before and after crosslinking. The heating rate during the analysis was set at 10°C/min.

### Scanning electron microscopy

The surface and cross-section of the atrial patch were characterized using scanning electron microscopy (SEM) (EM-30 +, COXEM, South Korea) at an accelerating voltage of 15 kV. The cross-sections were exposed by fracturing the specimens after being frozen in liquid nitrogen, coated with Au and observed under SEM at a magnification of 1000.

### Inverted fluorescence microscope

Andr (0.4 mg/mL) and RITC (0.2 mg/mL) were dissolved in acetone. The solution was stirred in the dark at 25°C for 12 h [22]. Subsequently, the acetone solution was evaporated under vacuum. Because unreacted RITC dissolves in water, deionized water was added to the product. The solution was sonicated, centrifuged and removed. Finally, the solution was freeze-dried to obtain RITC-labeled Andr. The dried RITC-labeled Andr was dissolved in acetone, and using the aforementioned method, drug-loaded patches were fabricated. The distribution of the RITC-labeled Andr within the patch was observed under an inverted fluorescence microscope (Ti2-E, Nikon, Japan).

### Mechanical property

The atrial patch was cut into dumbbell shapes, and the mechanical properties of the atrial patches were determined using a Universal Testing Machine (UTM2102, Sansi, China) according to GB/T 1040.1-2018. The tensile tests were conducted at a speed of 50 mm/min. The stress-strain curve was recorded until the failure of the sample, with which the tensile modulus was calculated.

To investigate its mechanical properties at 37°C, we used Dynamic Mechanical Analysis (DMA, Discovery DMA 850). The test was performed in tensile mode with an amplitude of 50 μm and a frequency of 1 Hz. The test temperature range was from −20°C to 50°C, and oscillation Temperature Sweep was performed with a temperature increment of 3°C and a 1 min soak time.

### Water contact angle

The hydrophilicity of the atrial patch was evaluated using a contact angle goniometer (Theta Flex, Biolin, Sweden) to measure the static water contact angle (WCA) with a 2 μl water droplet as the probe liquid. Each sample was measured at least three times to ensure the accuracy and reliability of the results.

### Degradation rate

The atrial patches (2 × 2 cm^2^) was weighed at 25°C (*W_D0_*), then immersed in a PBS solution at 37°C and shaken at a speed of 100 rpm (*n* = 3). The samples were removed and weighed at 1 and 2 weeks, rinsed three times with deionized water, and then, weighed again (*W_D_*_1_) after being freeze-dried for 1 day. The PBS was replaced weekly. The formula for calculating the remaining mass percentage (%) was *W_D_*_1_/*W_D_*_0_ × 100%.

### Swelling studies

The atrial patches (2 × 2 cm^2^) were weighed at 25°C (*W_S0_*), then immersed in a deionized water/PBS solution at 37°C for 12 h to allow for complete swelling. After removal from the solution, it was wiped with soft tissue to eliminate excess surface water before its weight (*W_S1_*) was measured. The patch was then returned to the solution. The weight of the patch was recorded hourly until the mass remained consistent for three consecutive measurements, signifying complete swelling. The swelling ratio *Q* was calculated from equation: *Q* = *W_S_*_1_/*W_S_*_0_ × 100%.

### Drug release *in vitro*

The *in vitro* drug release performance of the atrial patch containing Andr was evaluated using a UV–Vis spectrophotometer (UV-2600i, Shimadzu, Japan). Initially, a standard curve for Andr was obtained by correlating the drug concentration with its absorbance value at a fixed wavelength peak (226 nm) using the UV–Vis spectrometer. As Andr has limited solubility in a PBS solution, we first dissolved it in ethanol and subsequently prepared a ethanol/PBS solution (ethanol/PBS = 5/95 V/V).

The patch was immersed in 10 mL of PBS and placed inside a thermostatic bath shaker (THZ-103B, Shanghai Yiheng Instrument Co., Ltd) set at 37°C with a shaking speed of 100 rpm/min. At specific time intervals, 1 mL of the PBS was withdrawn and replaced with 1 mL of fresh PBS. Subsequently, the drug concentration in the withdrawn 1 mL of PBS solution was measured using UV–Vis spectroscopy, allowing the calculation of the total concentration in the 10 mL PBS solution to generate the drug release curve. Similarly, ethanol was added to the 1 mL test sample to prepare a ethanol/PBS solution (ethanol/PBS = 5/95 v/v). Dilutions were performed as necessary.

The drug release data were analyzed using the Korsmeyer–Peppas equation (Equation 1) [23]. The parameters of each equation were determined using a nonlinear least-squares fitting method.


(1) $$M_{t}/M_{\infty }=kt^{n},$$

where *M_t_* is the concentration of the drug released at time *t*, *M_∞_* is the concentration of the drug released at equilibrium, *k* is constants incorporating the structural and geometric characteristics of the patch and *n* is the release exponent describing the mode of the transport mechanism. When *n *= 0.5, it is a pure diffusion process (Fickian diffusion); when *n *= 1, it represents the Case II transport mechanism; when 0.5 < *n  *<  1, it is the result of Fick diffusion and polymer relaxation.

### Ethics statement

The *in vivo* experiments were authorized by the Scope Research Institute of Electrophysiology Application for Laboratory Animal Welfare and Ethical Review (202110 Edition) (IACUC Issue number: SGLL20230902172).

### Building the SP model

Adult male Sprague–Dawley rats weighing 200–250 g were used in this study. SP rats were established as previously described [18, 24, 25]. Briefly, rats were anesthetized with sodium pentobarbital (i.p.; 60 mg/kg). Adequate anesthesia was confirmed by the absence of reflexes, and the atria were exposed through the left second intercostal space. After the pericardiotomy, sterile talcum powder was evenly dusted onto the left atrial surface. The Andr-loaded or blank-loaded patches were placed on the surface. Sham-operated rats underwent the same procedure without pericardiotomy or patch setting. Rats were randomly divided into the Sham group, SP model group (SP group), The Andr-loaded patches implantation group (Andr + SP group) and the blank-loaded patches implantation group (Blank + SP group).

### *In vivo* invasive electrophysiology operation

Three days postoperatively, an invasive electrophysiological operation was performed under anesthesia, as aforementioned [16]. The surface ECG parameters were manually calculated from the four limb leads. After exposing the one-sided jugular vein, a 1.1-French 8-pole electrode (Millar, 840-8214) was placed in the venous system up to the high right atrium site. Successful AF induction was defined as a period of rapid irregular atrial rhythm with an f wave and an absolute irregular RR interval lasting for at least 1 s. AF inducibility was determined by calculating the number of AF episodes versus the total number of procedures [26, 27].

### Hearts isolation, Langendorff-perfusion and electrical mapping preparations

After heparinization (0.5 mL/1000 g), all experimental rats were anesthetized with isoflurane for several minutes and subjected to cervical dislocation. After thoracotomy, hearts were promptly transferred to a Langendorff perfusion device, and the heart beats were recovered with the reformative Tyrode’s solution (119 mM NaCl, 25 mM NaHCO_3_, 4 mM KCl, 1.2 mM KH_2_PO_4_, 1 mM MgCl_2_, 1.8 mM CaCl_2_·2H_2_O and 10 mM D-glucose). Langendorff-perfusion system was equilibrated with 5% CO_2_ and 95% O_2_ with a flow velocity of 10 mL/min at 36.5°C. After observation of stable heartbeats for 8 min, isolated hearts were transferred to the electrical and optical mapping systems. A 15-min electrical recording was performed to monitor the cardiac rhythm and troubleshoot the potential perfusate stasis using two 64-channel multielectrode arrays (MEAs, EMS64-USB-1003, MappingLab Ltd., UK), as previously mentioned [28].

### Optical mapping for *ex vivo* hearts

First, 60 μl of blebbistatin (17 mM) was injected into the perfusion opening of 11 min to cause the electromechanical uncoupling. Then, 50 μl F127 and 50 μl Rhod2-AM (3.6 mM) were respectively infused to enable the dye combinations to cytomembrane and cytoplasmic Ca^2+^ at 36.5°C. Two 530 nm light-emitting diodes (LEDC-2001, MappingLab Ltd) were employed to generate the fluorescence light disposed by the bandpass filter at wavelengths of 530 ± 20 nm. The excitation light was passed through a 550-nm long-pass filter and a dichroic mirror with a cutoff value of 638 nm. Light wavelengths above 638 nm were denoised using a 700-nm long-pass filter and then captured by the recording camera for voltage signals. Meanwhile, lights below 638 nm were winnowed by a 585 ± 40 nm bandpass filter and then imaged (OMS-PCIE-2002, MappingLab Ltd) to obtain the calcium signals. The sampling area for the atrium was a 16 × 16 mm square, with a spatial resolution of 128 × 128 pixels.

### Atrial electrophysiological evaluation protocol

All programmed pulses were released at a 1.5 × amplitude of the basic pacing threshold and a 2-ms pulse width. A steady S1S1 interval at 360 ms was utilized to evaluate the action potential duration (APD) and Ca^2+^ transient duration (CaD). A descending S1–S2 mode was used to delineate the recovery capability of cytoplasmic Ca^2+^ transients (CaT) at different S2 intervals from 100 to 50 ms with a basic cycle length (BCL, S1S1 = 167 ms). Another incremental S1–S2 mode was adopted to depict the spatial Ca^2+^ transient restitution curve at increased S2 intervals from 100 to 300 ms at a 25-ms step interval, according to the previous protocol [29]. The conduction properties were acquired using an S1–S1 pattern with the CL reduced from 240 to 160 ms at a 20-stepwise declined interval. AP and CaT alternans were measured using stepwise S1S1 stimuli, starting from 120 ms BCL and reducing to 70 ms BCL with 30 repeats. Every two S1S1 interval reductions were followed by a 2-s pause. Finally, atrial arrhythmias and re-entry phenomena were induced using a burst pacing protocol with variable pulses for every 50 stimuli at a decreased S1S1 interval from 50 to 20 ms with a 20-ms step time.

### Immunofluorescence analysis

Frozen sections were equilibrated to room temperature for 20 min, fixed with 4% PFA for 15 min, washed by PBS thrice and blocked by QuickBlock™ Blocking Buffer for Immunol Staining (P0260, Beyotime, China) at room temperature for 15 min. After blocking, primary antibodies were diluted in QuickBlock™ Primary Antibody Dilution Buffer for Immunol Staining (P0262, Beyotime, China) at 4°C overnight. The slides were then washed three times with PBS and incubated with the secondary antibody at room temperature for 60 min. The slides were sealed with anti-fluorescence quenching tablets and observed under confocal laser scanning microscopy. The primary antibodies are as follows: Anti-CD68 (MCA341, Bio-Rad, dilution 1/200) and Anti-iNOS (ab15323, Abcam, dilution 1/100).

### HL-1 cell culture

HL-1 atrial myocytes (purchased from SIGMA) were cultured in Claycomb medium, which was supplemented with 10% FBS, 100 U/mL penicillin, 100 μg/mL streptomycin, 0.1 mM norepinephrine and 2 mM L-glutamine.

### Bone marrow-derived macrophage cell culture

Bone marrow-derived macrophages (BMDMs) were isolated from 8-week-old male C57BL/6 mice using PBS flushing their femurs and tibias with PBS after stripping. The bone marrow cells were resuspended in DMEM containing 20% FBS and 100 ng/mL M-CSF. Cells were incubated for 7 days at 37°C and 5% CO_2_, with medium changed every 2–3 days. BMDMs were plated at a density of 1 × 10^6^ cells/cm^2^ and stimulated via 100 ng/mL LPS (L3012, SIGMA) and 20 ng/mL IFN-γ (285-IF, R&D System) for 24 h.

### ELISA

The supernatant was prepared according to the Mouse ELISA Kit for TNF-α (Multiscience, EK282/4-96).

### Cell viability assay

Cell viability was assessed using the Cell Counting Kit-8 (CCK-8) assay (Dojindo, Japan). HL-1 cells were seeded in 96-well plates at a density of 3000 cells/well. After stimulating, 10 μl CCK-8 was added to each well for 1–2-h incubation. The absorbance was measured at 450 nm using a microplate reader.

### DHE staining

The procedure for DHE staining has been described in our previous work [16]. In brief, according to the instructions (BestBio, China), the frozen sections were washed with washing buffer for 10 min, incubated with fluorescent dye at 37°C for 30 min, washed three times with washing buffer, mounted and imaged using fluorescence microscopy. For HL-1 cells, the procedure involved incubating with fluorescent dye for 30 min at 37°C in darkness, followed by three washes with PBS to eliminate background fluorescence, and finally, imaging by fluorescence microscopy.

### 5,6-Carboxy-2′,7′-dichlorofluoresceindiacetate staining

ROS levels in HL-1 cells were measured via 5,6-carboxy-2′,7′-dichlorofluoresceindiacetate (DCFH-DA) staining. Cells were incubated with DCFH probes for 30 min at 37°C in the dark, washed with PBS three times, digested by trypsin for 2 min at 37°C, centrifuged, resuspended and analyzed using flow cytometry. ROS levels in *BMDMs* cells were measured by fluorescence microscopy after incubating with DCFH probes.

### Mitochondrial ROS production detection

To measure mitochondrial ROS production, the HL-1 cells were stained with MitoSOX Red (Invitrogen) and MitoTracker Green (Invitrogen) after stimulation. Cells were imaged using a fluorescence microscope.

### Cytoplasmic and mitochondrial calcium ionic concentration detection

HL-1 cells were incubated with Rhod2 (4 µM) and Mitotracker Green (100 nM) to detect mitochondrial calcium ionic concentration or Fluo4 (5 µM) to detect cytoplasmic calcium ionic concentration for 15 min at 37°C, after which they were washed to Hanks Balanced Salt Solution and imaged via fluorescence microscopy.

### RNA extraction and quantitative reverse transcription-PCR

Total RNA was extracted from atrial tissues and HL-1 cells using TRIzol reagent (Invitrogen, Carlsbad, CA, USA) according to the manufacturer’s instructions. RNA concentration was quantified using a NanoDrop spectrophotometer and Agilent 2100 bioanalyzer (Thermo Fisher Scientific, MA, USA) and reverse-transcribed into cDNAs using PrimeScript RT (Takara, Japan) in accordance with the manufacturer's instructions. Quantitative reverse transcription PCR was performed using a PCR mixture (11203ES08; Yeasen, China) on a LightCycler 480II system (Roche). The following primer sequences were used:

GAPDH (forward, 5′‐GACATCAAGAAGGTGGTGAAGC‐3′, reverse, 5′‐TGTCATTGAGAGCAATGCCAGC‐3′),

TNF-α (forward, 5′‐CCCAGACCCTCACACTCAGATCAT‐3′, reverse, 5′‐GCAGCCTTGTCCCTTGAAGAGAA‐3′),

IL-1β (forward, 5′‐CTCTGTGACTCGTGGGATGATG‐3′, reverse, 5′‐CACTTGTTGGCTTATGTTCTGTCC‐3′),

NQO1 (forward, 5′‐GCTGCAGACCTGGTGATATT‐3′, reverse, 5′‐ACATGGTGGCATACGTGTAG‐3′),

HMOX1 (forward, 5′‐AGCATGTCCCAGGATTTGTC‐3′, reverse, 5′‐TCACCAGCTTAAAGCCTTCC‐3′),

### Western blotting

Total protein was extracted using RIPA lysis buffer (FDBio Science, Hangzhou, China) with PMSF for 10 min and quantified using a BCA Protein Assay Kit (YEASEN, China). A Precast Protein Plus Gel (36250ES10, YEASEN, China) was used. The bands were incubated with primary antibodies for 1.5 h at room temperature. The primary antibodies used in this study are as follows:

GAPDH (66240-1-Ig, Proteintech, at 1/5000 dilution in 5% BSA), HMOX1 (10701-1-AP, Proteintech, at 1/1000 dilution in 5% BSA), NQO1 (11451-1-AP, Proteintech, at 1/1000 dilution in 5% BSA), SOD1 (GTX100554, GeneTex, at 1/1000 dilution in 5% BSA) and SOD2 (GTX116093, GeneTex, at 1/1000 dilution in 5% BSA).

### Statistical analysis

Statistical analyses were performed using GraphPad Prism 8.0.1. Data were presented as mean ± SD. Differences between groups were compared using ANOVA test. No samples or results were excluded from the analysis. Statistical significance is indicated as follows: *P < 0.05, **P < 0.01 and ***P < 0.001. Graphs were generated using the GraphPad Prism (version 8.0.1) or FlowJo (V10) software.

## Results

### Synthesis of PEL triblock copolymers

**Figure 1.**
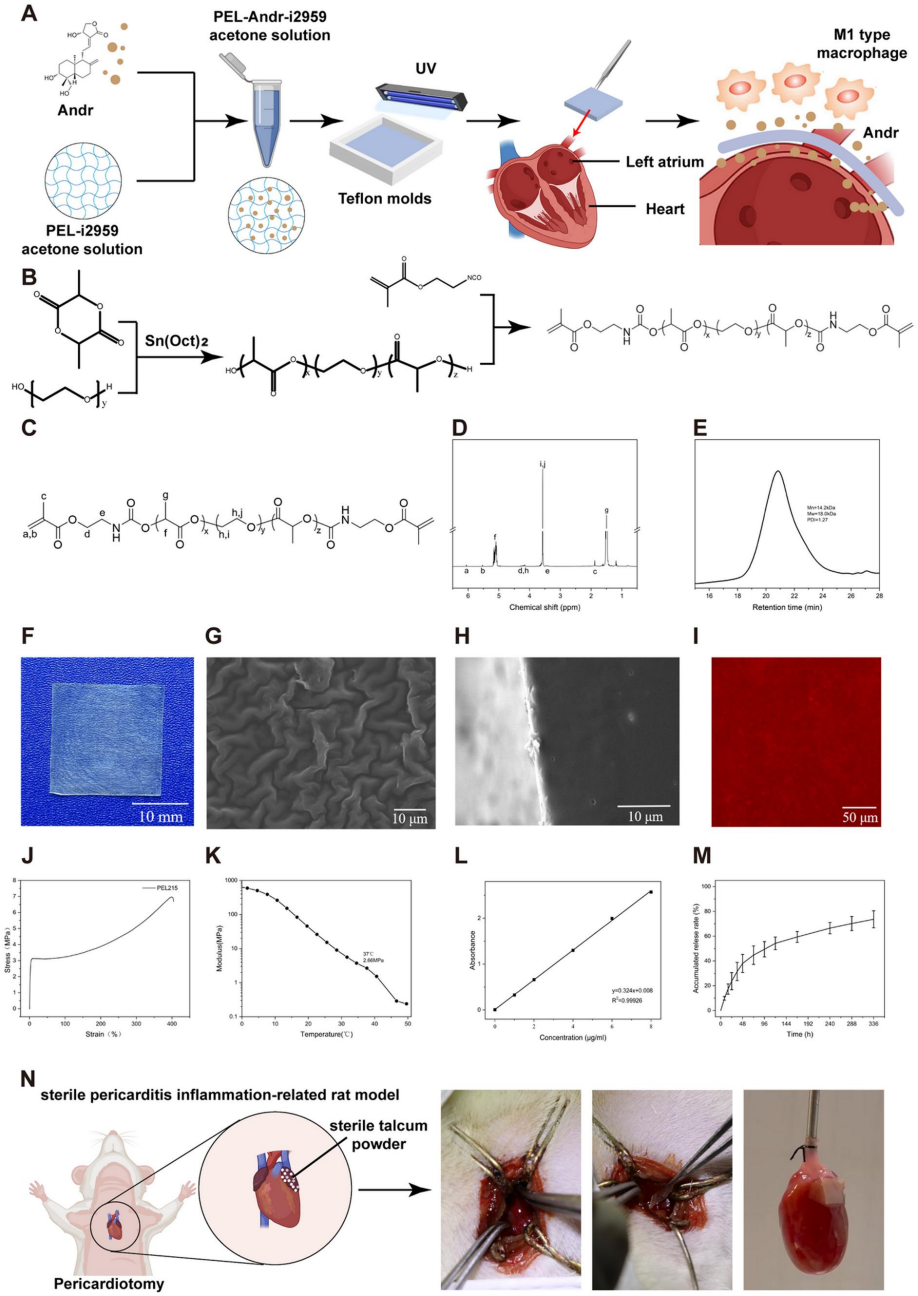
Fabrication of the patch. (**A**) Schematic illustration of patch fabrication and application. (**B**) Synthesis of PEL triblock copolymers and characterization of physical properties detection. Fabrication of drug-loaded atrial patch synthesis. ^1^H-NMR spectrum (**C, D**) and GPC spectrum (**E**) of PEL triblock copolymers. (**F**)The digital photo of the atrial patch. (**G, H**) Surface cross-sectional and SEM micrographs of the atrial patch. (**I**) The fluorescence microscopy image of the distribution of RITC-labeled Andr within the patch. (**J**) The stress–strain curve of the atrial patch. (**K**) Modulus of the atrial patch at different temperatures (DMA). (**L**) Concentration standard curve for Andr at a fixed wavelength peak (226 nm). (**M**) *In vitro* drug release profiles of Andr-loaded atrial patch. (**N**) Schematic diagram of the rat model and the patch attached to the left atrium. Retrieve from https://app.biorender.com/biorender-templates.

Figure 1A and B illustrates the synthesis of the PEL triblock copolymers. The composition, molecular weight and molecular weight distribution of PEL were characterized using ^1^H-NMR and GPC. The characteristic peak at 4.25 ppm (Figure 1D, H) corresponded to the -CH_2_- group linking the PEG segment to the PLA segment from the PEG fragment, confirming the successful construction of the PEL triblock copolymer. The peak integrals of -O-CH2-CH2- (3.65 ppm, Figure 1D, I and J) from the PEG segments were compared with peak integrals of -CH- (5.16 ppm, Figure 1D, F) The composition of the synthesized PEL copolymer was calculated from the PLA segments. Furthermore, characteristic peaks at 5.60 and 6.12 ppm indicate successful termination with the methacrylate functional group [30]. Using these peak integrals as references, the actual percentages of double-bond terminations were calculated. The results are summarized in Table 1. GPC curves for the PEL triblock copolymer revealed a narrow molecular weight distribution with a polymer dispersity index of 1.27. These collective results confirm the successful synthesis of the PEL triblock copolymer.

**Table 1.** Molecular weight and distribution of the PEL triblock copolymer

Mn(NMR)（kDa）	Mn(GPC)（kDa）	Mw(GPC)（kDa）	Sealing Rate（%）	PDI
14.3	14.2	18.0	48.1	1.27

### Fabrication of drug-loaded atrial patch

The patch, obtained from a Teflon mold through the casting method, was cut into 20 × 20 mm^2^ parts with a thickness of 0.161 ± 0.012 mm (Figure 1A and F). SEM was used to observe the surface morphologies and cross-sectional structures of the patches. The SEM images showed unevenness on the surface of the material, as shown in Figure 1G, which could be attributed to slow drying at room temperature during the casting process. As shown in Figure 1H, no significant pore structure was observed in the cross-sectional image, indicating the compact interior of the patch. The fluorescence microscopy images showed that red fluorescence was evenly distributed in the patch, indicating a uniform distribution of Andr (Figure 1I). Therefore, we successfully constructed a relatively homogeneous and structurally dense drug-loaded atrial patch.

### Characterization of physical properties of atrial patch

To understand the state of the PEL at 37°C, we conducted tests using DSC. During the second heating scan in the DSC test, the glass transition temperature (*T_g_*) of the crosslinked PEL was observed at 15.76°C, which is higher than that of the non-crosslinked PEL (9.59°C). This indicates that the patch exhibits a highly elastic state at 37°C. Furthermore, no crystallization peak was observed during the scanning process, which can be attributed to the use of DL-lactide during the synthesis [31].

The patch was specifically designed for direct implantation into the atrium after surgery, which requires specific mechanical properties to facilitate surgical manipulation. The obtained stress–strain curve (Figure 1J) from the tensile test indicates that the patch is a stiff and tough material at 20°C, with a Young’s modulus of 158.07 ± 23.82 MPa, yield stress of 3.48 ± 0.80 MPa and fracture elongation of 292.43 ± 35.42%. This provides benefits during clinical surgery by allowing easier manipulation. Furthermore, we used to measure the modulus of the patch as the temperature increased. As shown in Figure 1K, it was observed that the modulus rapidly decreased, reaching only 2.66 MPa at 37°C. At this temperature, the patch exhibited flexibility, enabling it to adapt well to heart movements and deformations.

Hydrophilicity, an important feature of surface properties, has a significant impact on the biocompatibility of atrial patches. WCAs were measured to determine the hydrophilicity of the patches. Given the addition of the PEG segment, the hydrophilicity of the material was enhanced, resulting in a decreased contact angle of PEL (95.65 ± 0.15°), which was significantly lower than that of PLA (131°) [32]. In addition, the contact angle gradually decreased over time, indicating water absorption by the material. This suggests that the PEL exhibited favorable wetting properties [33], which facilitated close contact of the patch with the atrial surface.

To explore the swelling conditions of the PEL patch, we calculated the swelling ratio Q in different solutions. The patch exhibited swelling ratios of 117.86 ± 1.10% in deionized water and 114.71 ± 0.93% in PBS. These results suggest a relatively low degree of swelling, indicating minimal volume change in the patch after swelling.

The biodegradability of atrial patches is crucial because nondegradable patches can lead to graft thickening, thereby increasing the risk of immunogenicity and disease transmission [34]. The presence of PLA fragments in this material confers biodegradability to atrial patches. The ester bonds within the patches were randomly hydrolyzed and cleaved in phosphate-buffered saline (PBS) [23]. After 14 days of degradation, the remaining mass of the PEL patch was 89.21 ± 1.60%, indicating a slight weight loss. However, under the influence of enzymes or other factors [35], the degradation rate of the patch *in vivo* was significantly higher than that *in vitro*.

### Drug release *in vitro*

The Andr release behaviors of the patches were then investigated in PBS solution based on the concentration standard curve for Andr at 226 nm wavelength (Figure 1L). As shown in Figure 1M, nearly 37.93% of the Andr was released in 48 h and 73.62% over 14 days.

We analyzed the drug release data using the Korsmeyer-Peppas equation to investigate the kinetics of drug release. Within the initial 48 h, a relatively rapid drug release was observed. The calculated value of the exponent “*n*” was 0.756, indicating a non-Fickian diffusion mechanism characterized by the involvement of both polymer relaxation and drug diffusion [36]. This can be attributed to the relaxation of the polymer and the significant concentration difference, which drives drug release. As time progressed, the drug release rate decreased gradually. This can be attributed to the decrease in the concentration difference and the completion of polymer relaxation. During this stage, the dominant mechanism of drug release shifted towards Fickian diffusion, where diffusion becomes the primary factor influencing drug release. This indicates that PEL patches have the ability to slow drug release and that this material holds great potential in the field of drug delivery systems.

### Andr-loaded patch treatment reduces AF vulnerability *in vivo*

**Figure 2.**
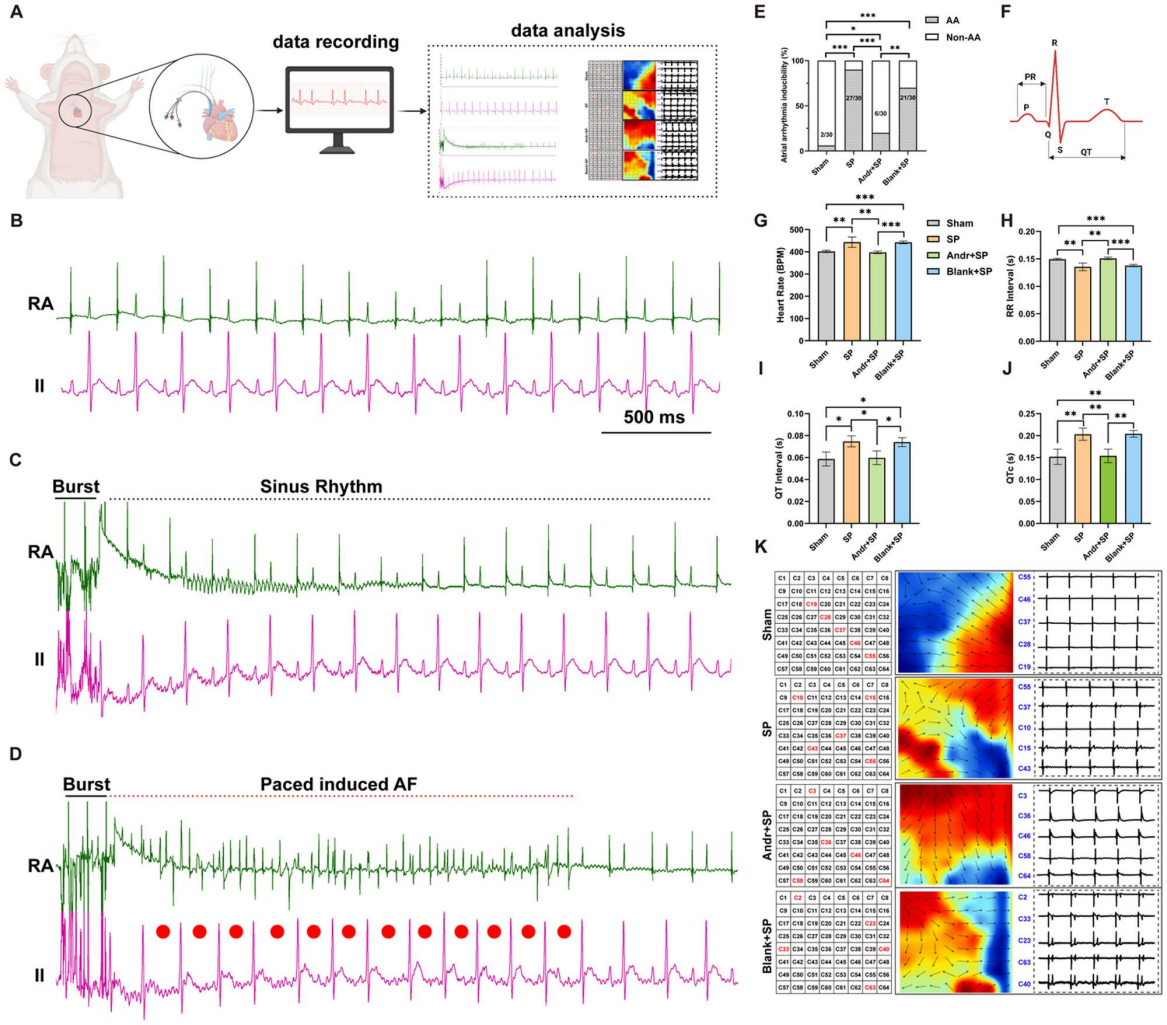
Andr-loaded patch treatment reduces AF vulnerability *in vivo*. (**A**) Diagram of invasive electrophysiological measurement and AF vulnerability detection *in vivo*. (**B**) Typical surface ECG (lead II) and intracavitary electrical signal recordings before burst pacing (in sinus rhythm). (**C, D**) representative simultaneous recordings of surface ECG (lead II) and intracardiac electrograms in rats without or with successfully induced AF. (**E**) Incidence of pacing-induced AF in rats (*n* = 6, each rat was measured 5 times). (**F**) Demonstration of various indicators of electrocardiogram. (**G**) Quantification of heart rate (*n* = 6). (**H**) Quantification of RR interval (*n* = 6). (**I**) Quantification of QT interval (*n* = 6). (**J**) Quantification of QTc (*n* = 6). (**K**) Representative electrical propagation heat map located on the left atrium. Data are shown as mean ± standard deviation (SD) and statistical analysis was performed with one-way ANOVA statistic test. *P < 0.05, **P < 0.01 and ***P < 0.001 indicate statistical significance between paired groups.

The surgical procedure for building the SP model is presented in Figure 1N. As reported in previous studies, the incidence of atrial arrhythmias peaks 2–3 days after cardiac or other noncardiac surgery; therefore, electrophysiological studies were performed on the third postoperative day. Invasive electrophysiological process is presented in Figure 2A. Typical surface ECG and intracavitary electrical signal recordings before and after burst pacing are shown in Figure 2B–D. Rats in the SP model group developed AF after 30 s of pacing at 50 Hz with a voltage of 1 mV, whereas rats treated with the Andr-loaded patch did not exhibit sustained AF after burst pacing. The AF inducibility was significantly elevated in the SP- and blank-loaded patch groups after burst pacing. However, the Andr-loaded patch exerted an effective anti-arrhythmic effect by reducing AF susceptibility (Figure 2E).

The QT interval prolongation reflects delayed cardiac repolarization, abnormal electrocardiography and is usually closely related to increased sensitivity to arrhythmia. Noninvasive ECG monitoring showed that SP rats had quicker heartbeats (Figure 2G) together with the shorter RR interval (Figure 2H), longer QT interval (Figure 2I) and longer QTCs (Figure 2J) than the Sham group rats. Treated with Andr-loaded patches could effectively relieve the above ECG changes. ECG analysis results were consistent with the previous results of invasive electrophysiological process inducing atrial fibrillation.

Apart from the invasive electrophysiological measurement and the ECG records, electrocardiogram activity also contains electrical conduction, mainly including conduction velocity and conduction heterogeneity. Thus, we performed the electric mapping on the left atrium *in vivo*. We found significant atrium conduction heterogeneity in the SP- and blank-loaded patch groups after burst pacing (Figure 2K). Considering the different heart rate above the four groups would influence the conduction velocity, we chosen the optical mapping technic to defect the conduction velocity *ex vivo* by Langendorff-perfusion system to reduce the impact of other factors such as heart rate. The results would be mentioned later.

### Effect of Andr-loaded patch on the atrial repolarization characteristic from the *V_m_* channel

**Figure 3.**
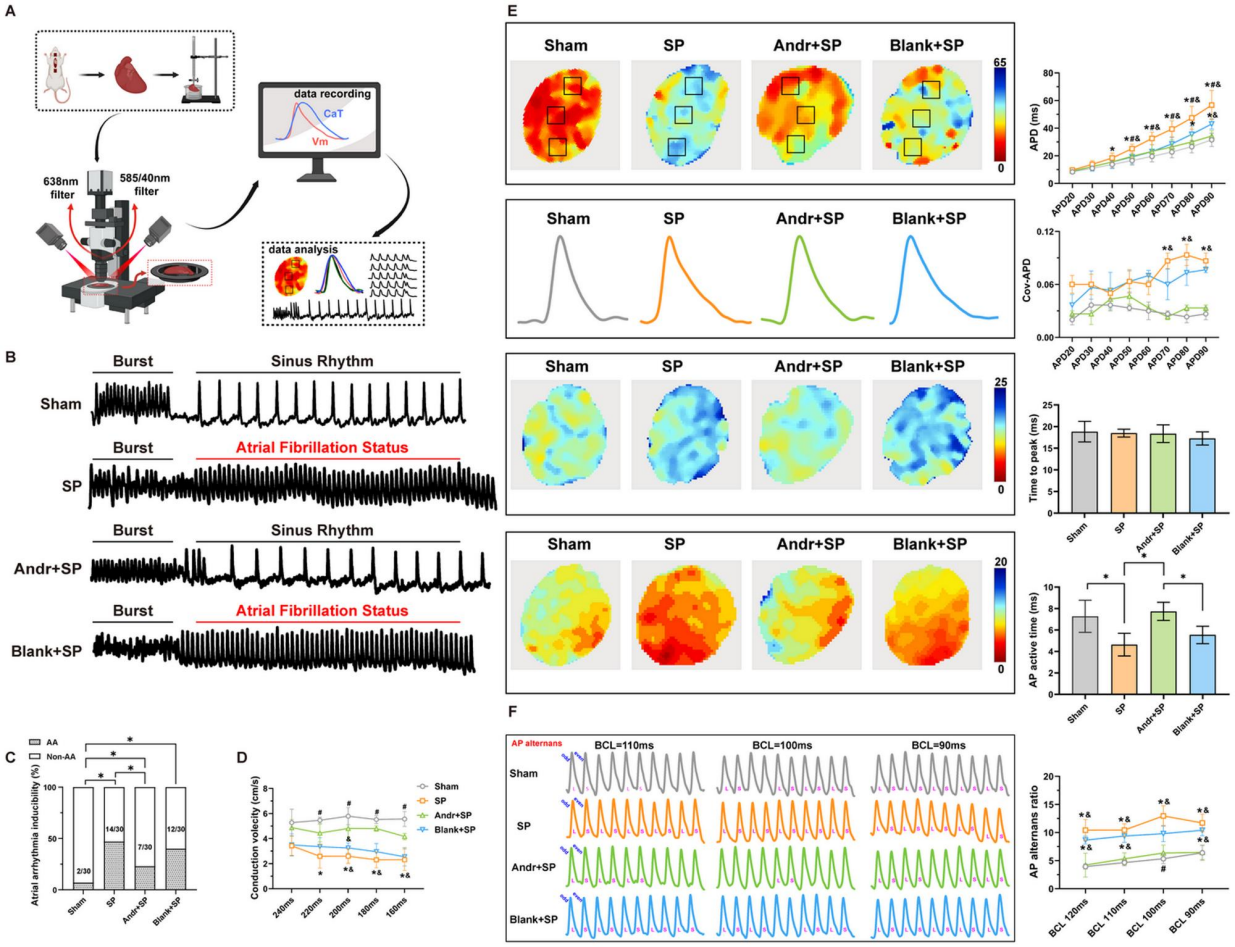
Effect of Andr-loaded patch on the atrial repolarization characteristic from the *V_m_* channel. (**A**) Diagram of the *ex vivo* optical mapping process. (**B**) Representative simultaneous recordings located on the left atrial epicardium without or with success fully induced AF. (**C**) The inducibility of atrial arrhythmia induced by burst pacing of four groups; *P < 0.05 indicates statistical significance between paired groups (*n* = 6, each rat heart was measured 5 times). (**D**) Quantitative statistics of atrial conduction velocities among different S1–S1 intervals from 240 ms to 160 ms, *P < 0.05 vs. Sham group; #*P* < 0.05 vs. blank-loaded group; &*P* < 0.05 vs. Andr-loaded group. (**E**) Representative heatmaps and trajectories of APD80 among four groups, *P < 0.05 vs. Sham group (*n* = 6); #*P* < 0.05 vs. blank-loaded group (*n* = 6); &*P* < 0.05 vs. Andr-loaded group (*n* = 6); representative time to peak maps and typical action time heatmaps on AP channel of four groups; quantitative analysis of APD differences from APD20 to APD90 and its COV, *P < 0.05 vs. control group (*n* = 6); &*P* < 0.05 vs. Andr-loaded group (*n* = 6); statistic analysis of peak maps on AP channel. *P < 0.05 indicates statistical significance between paired groups (*n* = 6). (**F**) Representative AP alternans among four groups at different S1S1 pacing intervals; quantitative calculations of AP alternans ratio among four groups, *P < 0.05 vs. Sham group (*n* = 6); &*P* < 0.05 vs. Andr-loaded group (*n* = 6); data are shown as mean ± standard deviation (SD) and statistical analysis was performed with one-way ANOVA statistic test.

The *ex vivo* optical mapping process is presented in Figure 3A. First, the inducibility of atrial arrhythmia was recorded using a burst-pacing protocol. As expected, both the inducibility of AF and atrial tachycardia increased substantially in the SP and blank-loaded patch groups, while patch intervention partly rescued atrial arrhythmia vulnerability (*P* < 0.05) (Figure 3B and C). Importantly, atrial CV, which did not be detected *in vivo* aforementioned, was significantly reduced in the SP and blank-loaded patch groups compared to Sham controls among different S1-S1 intervals from 240 ms to 160 ms, with the Andr-loaded patch partially restoring spatial conduction (*P* < 0.05). Then based on the basic pacing protocol, we described epicardial APD in three different areas *in situ*. The COV of the APD ratio was calculated using the standard deviation/mean of the three areas. As shown in Figure 3E, typical trajectories of APD90 were drawn for the four groups. For AP signals, the SP models showed increased durations from APD40 to 90 in comparison to the Sham and Andr-loaded patch groups, while the APD80 and APD90 of the blank-loaded groups showed no significant differences compared to the SP groups (*P* < 0.05). The patch containing andrographolide normalized the spatial differences from APD40 to 60 at the tissue level but could not rescue the COV-APD alterations from APD70 to 90 (*P* < 0.05). Furthermore, there were significant differences in peak and active times in the AP channels. Atrial active times of AP signals shortened synchronously in SP and blank-loaded models, while patch treatments reversed these changes (*P* < 0.05). The peak time values in the AP channel did not show significant differences across the four groups. To investigate the arrhythmogenic AP among different groups, we implemented a rapid pacing protocol. It was discovered that the AP alternans ratio increased in parallel in the SP and blank-loaded patch groups compared to the Sham controls and the Andr-loaded patch groups (*P* < 0.05). Typical alternating images are shown in Figure 3F.

### Andr-loaded patch treatment attenuated atrial inflammation *in vivo* and *in vitro*

**Figure 4.**
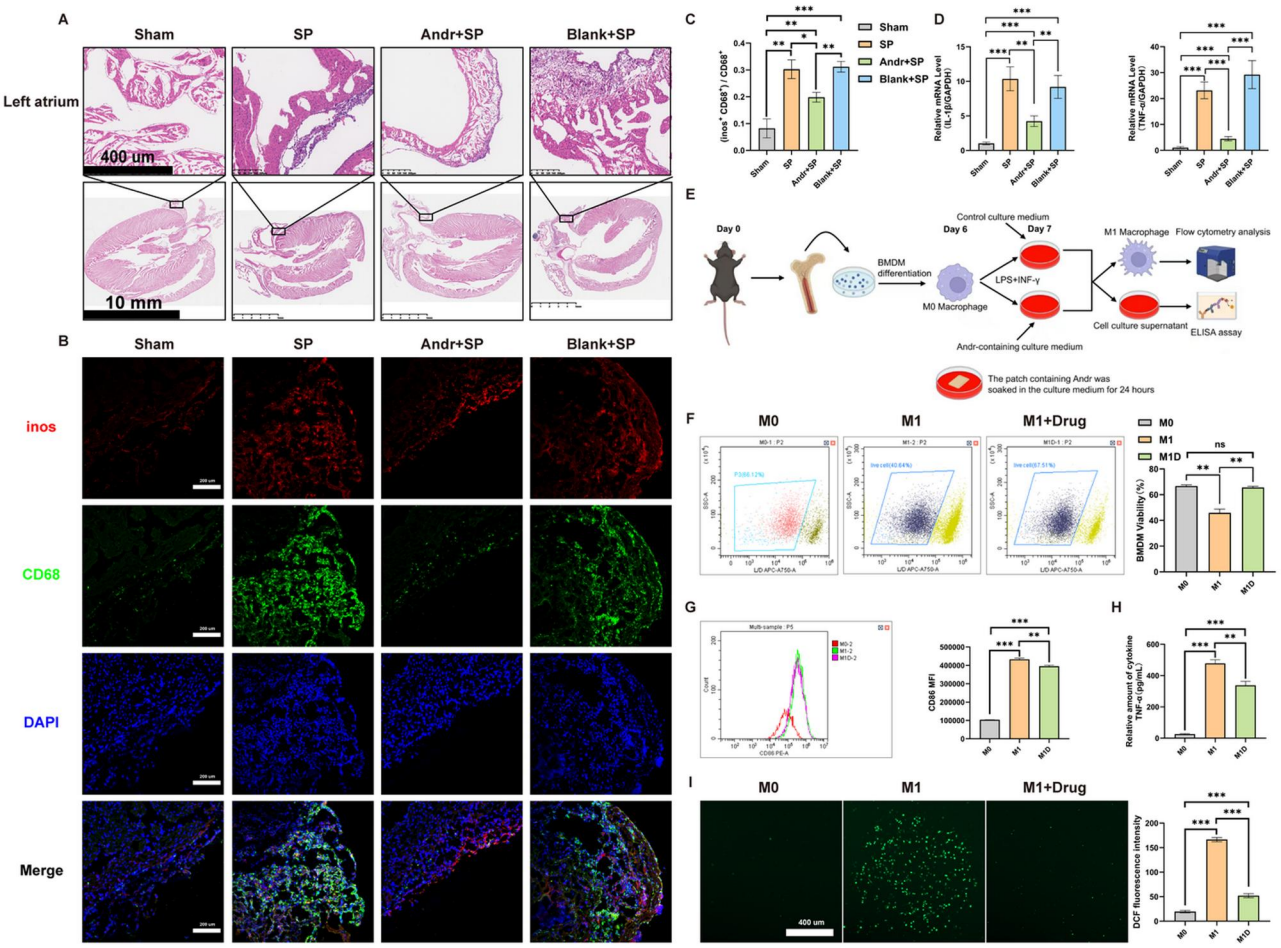
Andr-loaded patch treatment attenuated atrial inflammation *in vivo* and *in vitro*. (**A**) Representative images of hematoxylin-eosin (HE) staining. (**B**) Representative images of M1 macrophage polarization in the atrial of each group. M1 macrophage was labeled by inos positive together with CD68 positive. (**C**) Quantitative analysis of the proportion of M1 macrophage (*n* = 3). (**D**) Quantitative analysis of the expression of TNF-α and IL-1β (*n* = 3). (**E**) Diagram of the isolation and simulation of the BMDMs. (**F**) Representative images of propidium iodide (PI)-positive apoptotic BMDMs determined by flow cytometry. Quantitative analysis of the PI-positive apoptotic BMDMs (*n* = 3). (**G**) Representative images of the CD86-positive BMDMs determined by flow cytometry. Quantitative analysis of the CD86-positive BMDMs (*n* = 3). (**H**) TNF-α levels in BMDMs medium determined by ELISA (*n* = 3). (**I**) Representative images of ROS level in BMDMs using DCFH-DA probe (*n* = 3). Quantitative analysis of the DCF fluorescence density. Data are shown as mean ± standard deviation (SD), and statistical analysis was performed with one-way ANOVA statistic test. *P < 0.05, **P < 0.01 and ***P < 0.001 indicate statistical significance between paired groups.

Hematoxylin-eosin staining of SP rat left atrial tissues showed that Andr-loaded patch treatment significantly attenuated inflammatory cells attached to the surface of the atrium and caused less cardiac injury in either the atrium (Figure 4A) or ventricle (Supplementary Figure S1). Beyond that, we would like to test the macrophage polarization in local left atrium. To this part, we performed multiple fluorescence co-staining to assess the classification of macrophages. M1 macrophage (inos^+^CD68^+^) and M2 macrophage (Arg1^+^CD68^+^) were detected. Interestingly, after operation 3 days, we found that M1 macrophages significantly infiltrated into the epicardial myocardial tissue, while the endocardium remained unaffected (Figure 4B and C). And we found it hardly to observe M2 macrophages (Supplementary Figure S2). Immunofluorescence staining of TNF-α in the atrial tissues showed obviously increased TNF-α levels in the SP and blank-loaded patch groups, and this increase was largely diminished by the Andr-loaded patch treatment (Supplementary Figure S3). These results were further confirmed by measuring the mRNA levels of TNF-α and IL-1β (Figure 4D).

*In vitro*, BMDMs were isolated from 8-week-old male C57BL/6 mice. The Andr-loaded patch was soaked in 10 mL of DMEM for 24 h at 37°C, and the Andr-containing medium was collected. BMDMs were polarized to be M1 macrophages by simulation with LPS + IFN-γ as materials mentioned with the Andr-containing medium or not (Figure 4E). LPS + IFN-γ stimulation-induced apoptosis in BMDMs could be completely resolved by the Andr-containing medium treatment (Figure 4F). flow cytometry analysis confirmed the enhanced expression of CD86-positive M1 cells, which was slightly inhibited by treatment with Andr-containing medium (Figure 4G). In addition, when we examined the release of TNF-α, the result was similar to the flow cytometry analysis (Figure 4H). Finally, quantification of DCFH-DA fluorescence intensity showed that the increasing intracellular ROS levels in M1 cells, which could be reversed by treatment with Andr (Figure 4I).

### Andr-loaded patch treatment alleviated oxidative stress injury *in vivo* and *in vitro*

**Figure 5.**
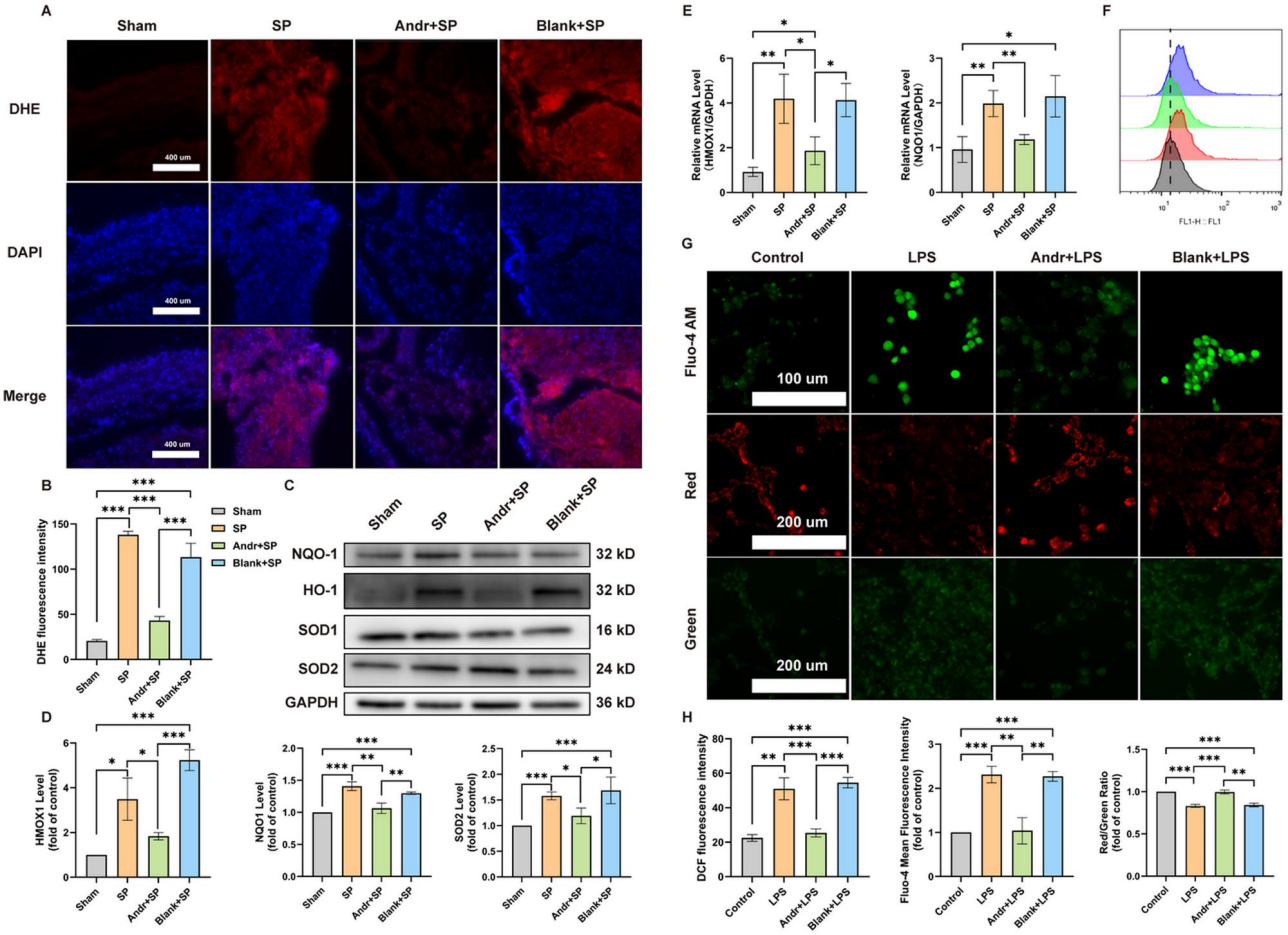
Andr-loaded patch treatment alleviated oxidative stress injury *in vivo* and *in vitro*. (**A**) Representative dihydroethidium (DHE) fluorescence images of superoxide production in rats. (**B**) Quantitative analysis of the fluorescence density of DHE (*n* = 3). (**C**) Representative blots of heme oxygenase-1 (HMOX1), NQO1, SOD1 and SOD2. (**D**) Quantitative analysis of the expression of HMOX1, NQO1 and SOD2 (*n* = 3). (**E**) qPCR was carried out to determine the mRNA levels of HMOX1, NQO1 (data were normalized to GAPDH, *n* = 3). (**F**) Representative images of DCFH-DA HL-1 cell determined by flow cytometry. (**G**) Representative Fluo4 fluorescence images of Ca^2+^ and JC-1 mitochondrial membrane potential (MMP) assay in HL-1 cells. (**H**) Quantitative analysis of the fluorescence density of DCF, Fluo4 and JC-1 (*n* = 3). Data are shown as mean ± standard deviation (SD), and statistical analysis was performed with one-way ANOVA statistic test. *P < 0.05, **P < 0.01 and ***P < 0.001 indicate statistical significance between paired groups.

As aforementioned, oxidative stress may significantly contribute to POAF, and in our previous research, Andr treatment alleviated oxidative stress injury in a rapid atrial pacing-induced rabbit acute AF model. We performed DHE staining to measure ROS in SP rat left atrial tissues and found that the level of ROS generation was significantly increased in both SP and blank-loaded patch groups compared to those in Sham controls and Andr-loaded patch groups (Figure 5A and B). As for NRF2/KEAP1 complex, we found that, compared to the Sham group, the green/red value was upregulated in the other three groups (Supplementary Figure S4). The expression levels of HMOX1, NQO1 and SOD2 (Figure 5C and D) were determined. Additionally, the mRNA levels of the antioxidant-related genes HMOX1, NQO1 in the left atrial tissues of SP rats were validated using RT-qPCR (Figure 5E). We found that the levels of NQO1 and HMOX1 were significantly increased in both SP and blank-loaded patch groups compared to those in Sham controls and could be effectively inhibited by Andr-loaded patch treatment. Although there was no significant difference in the expression of SOD1, we found that the expression of SOD2 was markedly increased in the Andr-loaded patch group compared to that in the SP and blank-loaded patch groups. SOD2, Superoxide Dismutase 2, mainly exerts function in mitochondrion. Thus, mitochondrion maybe crucial in the development of POAF and the effect of Andr.

To determine a suitable concentration for LPS stimulation, the viability of HL-1 cells was assessed using the CCK8 assay. Significant cytotoxicity was observed when treated with 1 µg/mL LPS for 24 h (Supplementary Figure S5), which was consistent with results obtained by others. Next, we assessed the effect of a medium containing various concentrations of LPS on cell death. Regardless of whether the treatment involved low, medium or high concentrations of Andr, the cell death induced by LPS could be effectively attenuated, and we found no significant cytotoxicity caused by the high Andr concentration medium treatment compared to the low or medium treatments (Supplementary Figure S6). Thus, high Andr concentration medium treatment and 1 µg/mL LPS were selected as the optimal drug concentration and stimulating concentration, respectively. DHE staining (Supplementary Figure S7) and quantification of DCFH-DA fluorescence intensity by flow cytometry showed that LPS promoted intracellular ROS levels, which could be reversed by treatment with Andr (Figure 5F). The levels of mitochondrial ROS were similar (Supplementary Figure S8). Finally, intracellular calcium ions and mitochondrial calcium content were detected using Fluo4 and Rhod-2 AM staining (Supplementary Figure S9). An increase in Fluo4 intensity was observed following LPS stimulation, which could be inhibited by the high Andr concentration medium treatment. Mitochondrial calcium accumulation, as measured by Rhod-2 AM staining and co-staining with MitoTracker Green in the LPS group, was significantly reduced compared to that in the control group. These results were confirmed using the JC-1 MMP assay (Figure 5G and H).

### Effect of Andr-loaded patch on the atrial calcium handling capabilities, the arrhythmogenic alternans and re-entry *ex vivo*

**Figure 6.**
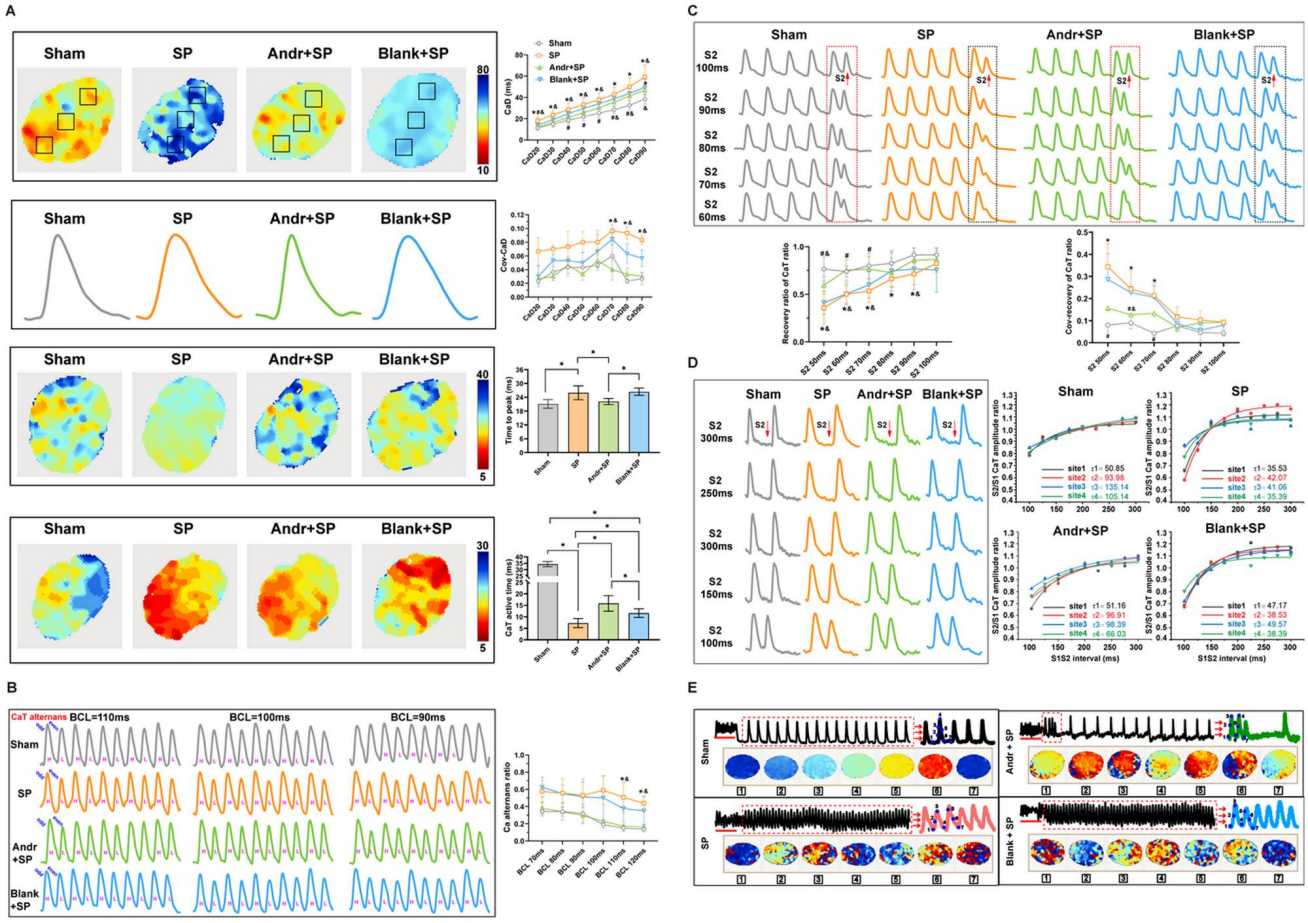
Effect of Andr-loaded patch on the atrial calcium handling capabilities, the arrhythmogenic alternans and re-entry *ex vivo*. (**A**) Representative heatmaps and trajectories of CaD80 among four groups, *P < 0.05 vs. Sham group (*n* = 6); #*P* < 0.05 vs. blank-loaded group (*n* = 6); &*P* < 0.05 vs. Andr-loaded group (*n* = 6); representative time to peak maps and typical action time heatmaps on Ca^2+^ channel of four groups; quantitative analysis of CaD differences from CaD20 to CaD90 and its COV, *P < 0.05 vs. control group (*n* = 6); &*P* < 0.05 vs. Andr-loaded group (*n* = 6); statistic analysis of peak maps on Ca^2+^ channel. *P < 0.05 indicates statistical significance between paired groups (*n* = 6). (**B**) Representative Ca^2+^ alternans among four groups at different S1S1 pacing intervals; quantitative calculations of Ca^2+^ alternans ratio among four groups, *P < 0.05 vs. Sham group (*n* = 6); &*P* < 0.05 vs. Andr-loaded group (*n* = 6); (**C**) Representative schematic diagrams of S2 induction (showed in the dashed box) after five consecutive S1 stimuluses in four groups; calculation of the recovery ratio of CaT ratio among four groups and its statistical analysis for the COV-recovery of CaT ratio, *P < 0.05 vs. Sham group (*n* = 6); #*P* < 0.05 vs. blank loaded group (*n* = 6); &*P* < 0.05 vs. Andr-loaded group (*n* = 6); (**D**) The acquirements of S2/S1 CaT amplitudes from the incipient 100 ms-S1S2 interval with a incrementally stepwise S1-S2 interval; quantitative statistics of the S2/S1 amplitude ratio among four different sites of atrium of each group, *P < 0.05 vs. Sham group (*n* = 6); #*P* < 0.05 vs. blank-loaded group (*n* = 6); &*P* < 0.05 vs. Andr-loaded group (*n* = 6). (**E**) Typical phase images of dynamic re-entry phenomenon on AP channel. Data are shown as mean ± standard deviation (SD), and statistical analysis was performed with one-way ANOVA statistic test.

We detected the Ca^2+^ in HL-1 cell before. So, how about the change of the Ca^2+^ in the whole left atrium? We performed optical mapping to verify the *in vitro* results *ex vivo.* Same as previous AP measurement, we described epicardial CaD in three different areas *in situ*. As shown in Figure 6A, typical trajectories of CaD90 were drawn for the four groups. In terms of CaT signals, both SP and blank-loaded patch groups prolonged the Ca^2+^ handling times in atrium as indicated from CaD40 to CaD90; meanwhile, heterogeneous calcium handle dynamics were also observed in both SP and blank-loaded patch groups when compared to control and patch curation groups, especially for the recovery points, including COV-CaD70, COV-CaD 80 and COV-CaD 90 (*P* < 0.05). Atrial active times of CaT signals shortened synchronously in SP and blank-loaded models, while patch treatments reversed these changes (*P* < 0.05). In both the SP and blank-loaded patch groups, local aseptic inflammation potentially prolonged the CaT duration (*P* < 0.05).

**Figure 7.**
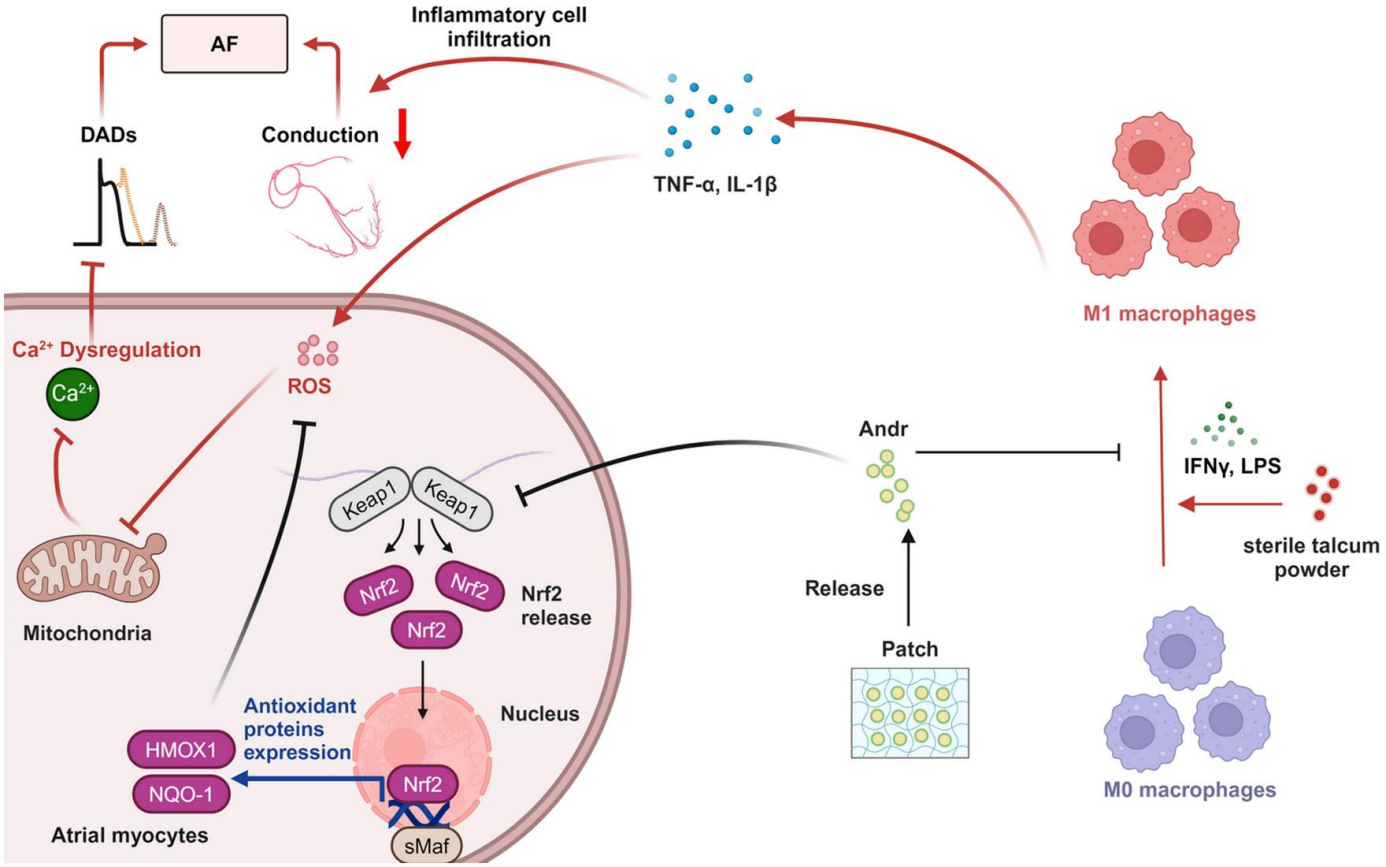
Therapeutic effect of Andr-patch on POAF by regulating the ROS balance and Ca^2+^. Retrieve from https://app.biorender.com/biorender-templates.

To investigate the arrhythmogenic CaT alternans among different groups, we implemented a rapid pacing protocol. It was discovered that the CaT alternans ratio increased in parallel in the SP and blank-loaded patch groups compared to the Sham controls and the Andr-loaded patch groups (*P* < 0.05). Typical alternating images are shown in Figure 6B.

At the intact tissue level, atrial calcium handling capability was evaluated by calculating the ratio of the maximum CaT amplitude induced by the inserted S2 to the CaT amplitude induced by the preceding S1. Evidently, the recovery of the CaT ratio decreased, and the COV recovery of the CaT ratio increased as the S1–S2 intervals shortened. Compared to the control group, Ca^2+^ handling stability and homogeneity significantly deteriorated in the SP and blank loaded groups, especially in the S2 beats at 50–70 ms BCL (*P* < 0.05). Furthermore, to unfold the spatial Ca^2+^ modulating dynamics, we portrayed the CaT restitution curve at four variable locations of the atrium. Typical recordings of programmed stimuli were displayed in Figure 6 (6C: recovery of the CaT ratio; 6D: CaT restitution curve). Corresponding to the aforementioned CaT recovery results, the spatial variability of CaT restitution kinetics was intuitively enlarged in both the SP and blank-loaded groups instead of the control and Andr-loaded patch groups.

Finally, the inducibility of atrial arrhythmia was recorded using a burst-pacing protocol (Figure 6E). As expected, both the inducibility of AF and atrial tachycardia increased substantially in the SP and blank-loaded patch groups, while patch intervention partly rescued atrial arrhythmia vulnerability (*P* < 0.05). Meanwhile, we observed the re-entry phenomenon accompanied by arrhythmia episodes through AP-phase videos, which corroborated one component of re-entrant arrhythmia in pathological experimental models.

## Discussion

POAF is a common postoperative complication in both cardiac and noncardiac surgeries, which significantly increases hospital stay, stroke risk and in-hospital mortality rates. Despite evidence linking inflammation and oxidative stress to POAF development, current treatments lack atrium-specific strategies and are hindered by dosage concerns and organ toxicity. Traditional Chinese medicine applications remain underexplored. The clinical potential of drug-loaded atrial patches for their biodegradability and mechanical properties has not been fully investigated in SP rat models, with limited preliminary research in beagles. Addressing this gap, we propose a novel approach integrating nanoparticle hydrogels with low-dose traditional Chinese medicine. This study aimed to validate the Andr-loaded atrial patch's preventative efficacy against POAF, potentially pioneering a novel therapeutic avenue.

Perhaps someone may ask “Why is a patch needed?” or “Is there a reason that Andr needs to be localized to the atrium for it to have its effect?” In fact, the difficulty in treating POAF patient in clinical practice lies in two points. First, we do not study the occurrence and development POAF very thorough. We do not know which specific inflammation to treat. This point is not the research direction of this study. Second, the issue of atrial blood supply and effective dosage of drugs when reaching the atrial site. Rapid blood flow and sparse epicardial blood supply make it difficult for drugs to exert functions as expected. This point is the research direction of this study actually. So, we simply designed a patch to local delivery the drug to atrium. Maybe someone may question that the design of the patch is too simple. Further research will be conducted on the adhesion and microenvironment response characteristics of the patch in future.

In this study, similar to postsurgical clinical outcomes, SP and blank-loaded patch groups displayed elevated heart rates, effectively lowered by Andr-loaded patch application. We chose a 1-s criterion for AF duration, aligning with previous findings and the early inflammatory phase characterized by fibrinous exudate. Andr-loaded patch treatment showed promise in reducing atrial arrhythmia *in vivo* and *ex vivo*. Optical mapping of *V_m_* and Ca^2+^ signals indicated Andr-loaded patches counteracted SP-induced APD and CaD elevations, suggesting a link between repolarization and intracellular calcium dysregulation. Spatial conduction analysis revealed significant CV reduction in SP and blank-loaded groups, with Andr treatment facilitating partial recovery. The S1–S2 protocol highlighted diminished calcium handling stability and uniformity in these groups, as shown by the CaT restitution curve and spatial variability in CaT restitution kinetics. AP-phase videos demonstrated re-entrant arrhythmias, highlighting re-entry’s role in arrhythmogenesis in this setup.

Growing evidence indicates that inflammation and oxidative stress are heavily implicated in POAF development. We observed that the use of Andr-loaded patches significantly ameliorated ROS generation, which significantly increased in the SP and blank-loaded patch groups. Increased expression levels of NRF2, HMOX1 and NQO1, together with increased mRNA levels, were observed. Notably, we found that Andr-loaded patches increased SOD2 expression, whereas there was no difference in SOD1 expression. This indicates that the role of mitochondria in this model may be critical. Consequently, we performed a series of *in vitro* experiments to verify mitochondrial dysfunction. First, DHE staining fluorescence and DCFH-DA fluorescence intensity detected by flow cytometry were used to measure intracellular ROS levels caused by LPS stimulation. The LPS stimulation of HL-1 cells directly leads to an increase in intracellular ROS production. A similar result was found in the ROS derived from mitochondria, which could be reversed via treatment with high Andr concentration medium. The JC-1 MMP assay was used to measure mitochondrial function. An imbalance in ROS is related to calcium-iron disorders. Therefore, Fluo4 and Rhod-2 AM staining were performed to measure intracellular calcium ions and mitochondrial calcium content. The increase in Fluo4 intensity and the decrease in Rhod-2 AM intensity were found in the LPS group, which could be reversed by high Andr concentration medium treatment, indicating a disorder of Ca^2+^ homeostasis. Interestingly, unlike others’ results published before, we did not find any significant fibrosis trend in either Masson staining, sirius red staining or WB experiment of type I collagen (Supplementary Figure S10). We speculated that under severe inflammation, cellulose seeped out of blood vessels and accumulated next to them, without forming large areas of fibrosis. It was quite common for adhesions to occur after clinical surgery.

With respect to the inflammation, HE staining and immunofluorescence staining showed the inflammatory infiltrate and the high expression of TNF-α in SP and blank-loaded patch groups, which could be largely diminished by the Andr-loaded patch treatment. When measuring the macrophage polarization condition, interestingly, unlike other results published before, we were pleasantly surprised to find that inflammatory cells infiltrated the epicardial surface without affecting deep areas such as the endocardium [24]. Significant CV reduction in SP and blank-loaded groups aforementioned might due to the infiltration of various immune cells. Regretfully, we did not conduct any relevant testing on Cx43. We further investigated the role of Andr in macrophage activation. BMDMs were polarized to be M1 macrophages by stimulation with LPS + IFN-γ, and apoptosis was induced during this process. We found that the Andr-containing medium could effectively inhibit LPS- and IFN-γ-induced apoptosis. However, the effect of Andr-containing medium on the polarization of macrophages shifting towards the M1 phenotype was slightly weak. We hypothesized that this weak pharmacological effect was due to an excessive LPS dose or a leaching solution with insufficient drug content. All the same, there remained some limitations of this study: (i) there remained some difference between humans and rats, such as the ion channel expression and distribution; (ii) we just applied the patch on the rats and further validation is still needed on large animals like beagle; (iii) we just verified the direct action of LPS on macrophages and HL-1 cells, respectively. The interaction between macrophages and atrial myocytes remained unclearly; and (iv) building this type of SP model needed to perform open chest surgery and there remains a large of other type of POAF patients clinically who underwent surgery for other parts.

## Conclusions

In summary, our results demonstrate that by regulating the inflammatory microenvironment, ROS balance and Ca^2+^ homeostasis, Andr-loaded atrial patch is effective for POAF in an SP rat model (Figure 7). This strategy could significantly reduce the drug using dosage and reduce liver and kidney side effects, thus, realizing the possibility of personalized healthcare. In the SP rat model, we better detect the therapy effect of Andr-loaded patches on Ca^2+^ mishandling and APD alternations. This study is a very meaningful attempt in POAF. It provides strong theoretical support for the prevention of POAF by local atrial administration, a new and previously untested medication strategy. Meanwhile, it provides a new treatment approach for other heart disease studies rather than cyclic administration. In addition, if ethics can pass through, larger scale clinical trials to validate the effectiveness and safety of treatment and the long-term effects of using such patches are highly necessary. Further research to improve the materials used for atrial patches, including their biocompatibility, biodegradability and mechanical properties, is needed to further enhance the effectiveness and safety of this treatment. Rather than the POAF, future research could make an attempt on other cardiac diseases research, such as AF and post myocardial infarction arrhythmias.

## Supplementary Material

rbaf040_Supplementary_Data
