# Supplementary material for: Enhanced prevention on postoperative atrial fibrillation by using anti-inflammatory biodegradable drug patch
Source: Regen Biomater. 2025 May 14;12:rbaf040. doi: 10.1093/rb/rbaf040 (PMC12202098; doi:10.1093/rb/rbaf040)
Supplement: rbaf040_Supplementary_Data [file rbaf040_supplementary_data.zip › Support Information.docx]

**Enhanced Prevention on Postoperative Atrial Fibrillation by Using Anti-inflammatory Biodegradable Drug Patch**

*Pengcheng Yu^1,2#^, Weiqi Lu^3#^, Huaxin Sun^4#^, Chengchen Huang^1,2^, Xiaolin Zhou^4^, Yuxing Wang^1,2^, Zhen Zhang^4^, Guosheng Fu^1,2^, Hanxiong Liu^4^*, Kefeng Ren^1,2,3^*, Xia Sheng^1,2^**

^1^ Key Laboratory of Cardiovascular Intervention and Regenerative Medicine of Zhejiang Province, Department of Cardiology, Sir Run Run Shaw Hospital, Zhejiang University School of Medicine, Hangzhou 310016, China

^2^ Engineering Research Center for Cardiovascular Innovative Devices of Zhejiang Province, Hangzhou 310016, China

^3^ MOE Key Laboratory of Macromolecular Synthesis and Functionalization, International Research Center for X Polymers, Department of Polymer Science and Engineering, Zhejiang University, Hangzhou 310058, China.

^4^ Department of Cardiology, The Third People’s Hospital of Chengdu, Affiliated Hospital of Southwest Jiaotong University, Chengdu 610014, China

# These authors contributed equally to this work.

* Corresponding to:

clin999@163.com (Hanxiong Liu)

renkf@zju.edu.cn (Kefeng Ren)

3101008@zju.edu.cn (Xia Sheng)


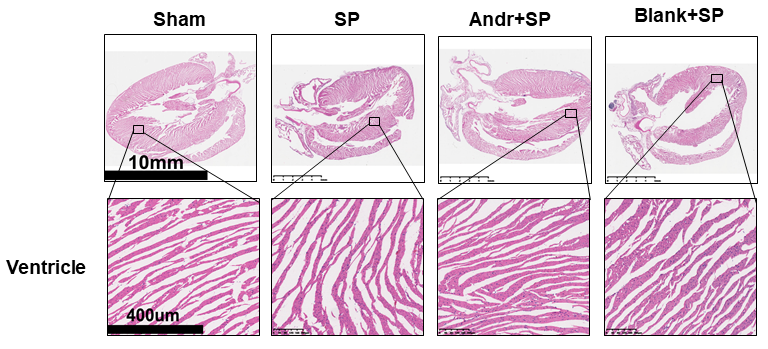


**Fig. S1.** Representative images of hematoxylin-eosin (HE) staining, in which we found no cardiac injury in ventricle.


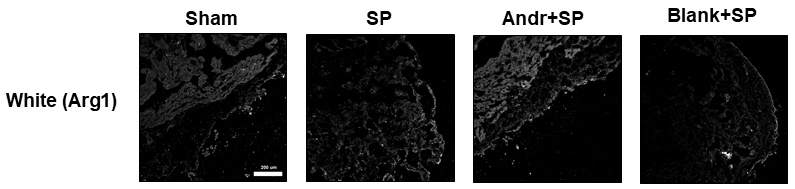


**Fig. S2.** Representative images of M2 macrophage polarization in the atrial of each group. M2 macrophage was labeled by Arg1 positive and presented as white color.


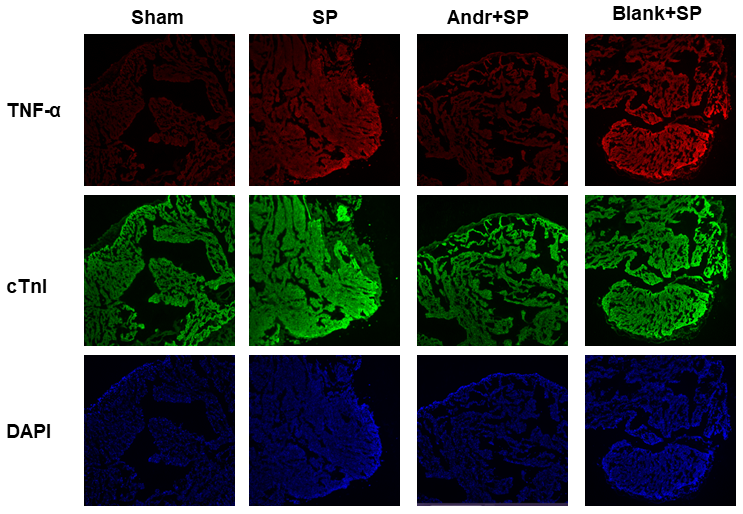


**Fig. S3.** Representative TNF-α and cTnI fluorescence images in rats’ tissues.


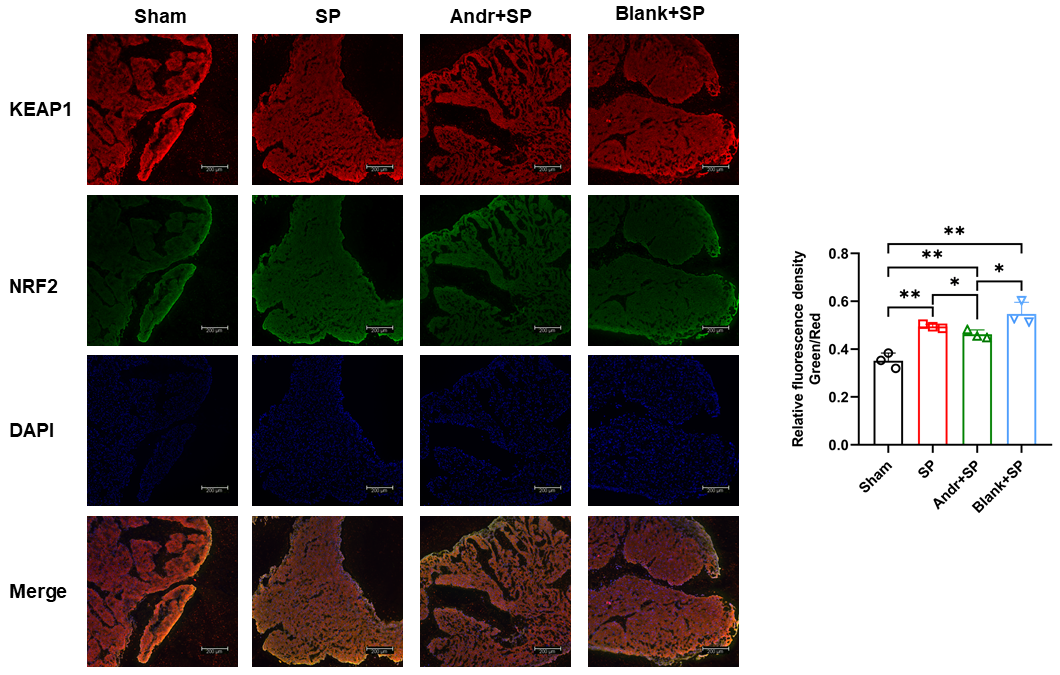


**Fig. S4.** Representative KEAP1 and NRF2 fluorescence images in rats’ tissues.


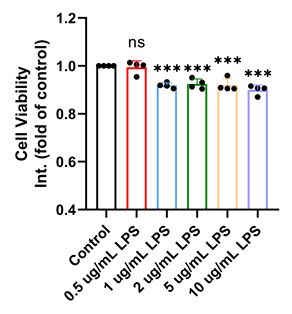


**Fig. S5.** To determine a suitable concentration for LPS stimulation, the viability of HL-1 cells was assessed using the CCK8 assay.


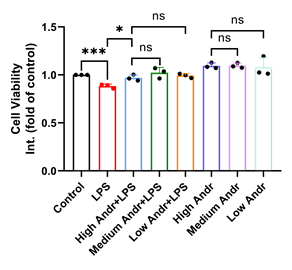


**Fig. S6.** To determine a suitable concentration for Andr-containing medium, the viability of HL-1 cells was assessed using the CCK8 assay.


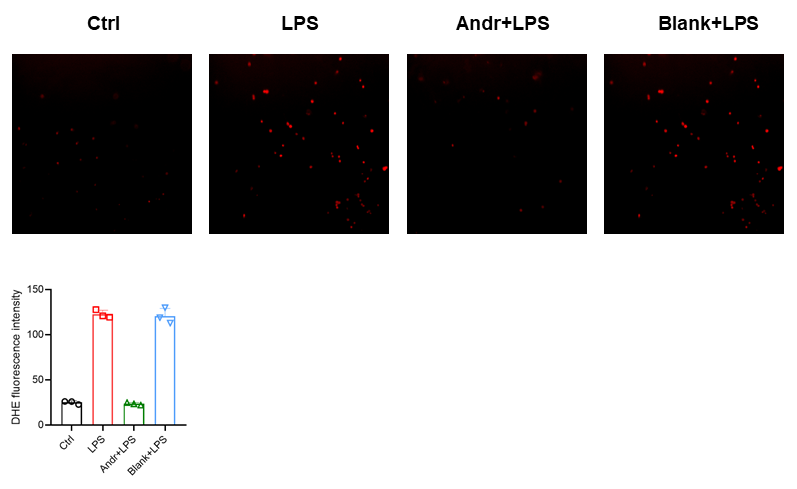


**Fig. S7.** Representative dihydroethidium (DHE) fluorescence images of superoxide production in HL-1 cells.


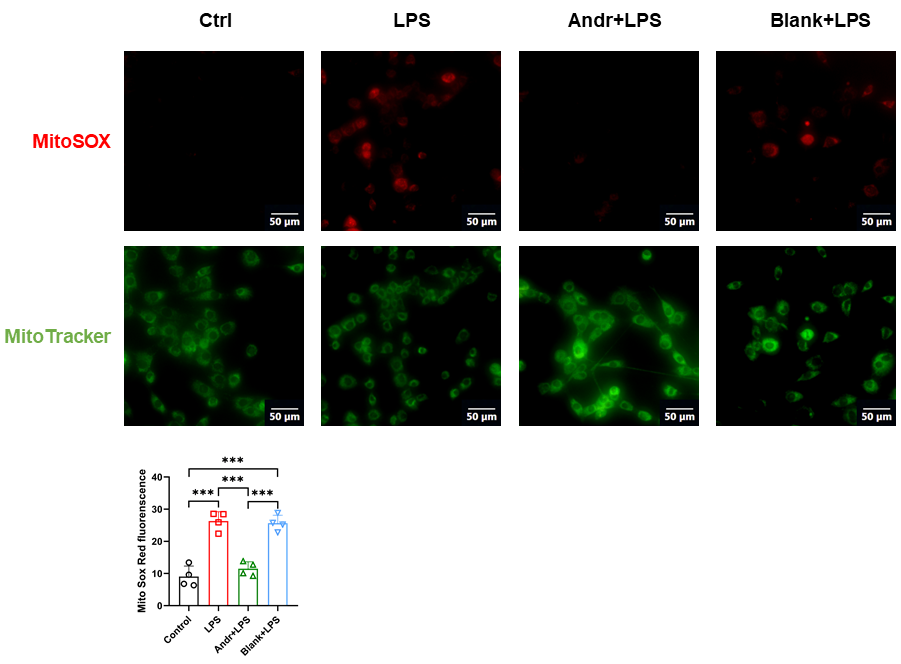


**Fig. S8.** Representative MitoSOX fluorescence images of mitochondrial ROS production in HL-1 cells.


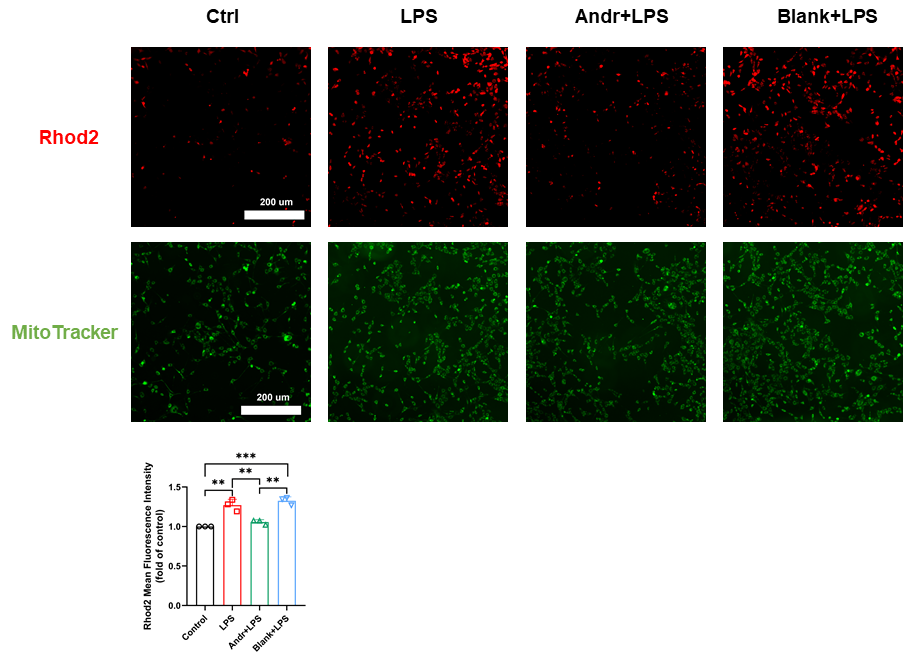


**Fig. S9.** Representative Rhod2 fluorescence images of mitochondrial Ca^2+^ in HL-1 cells.

**Fig. S10.** Representative Masson staining, sirius red staining and WB experiment of type I collagen.
